# An Assessment of Knowledge and Awareness Regarding Ozone Dentistry Among Dental Students at King Saud Bin Abdulaziz University for Health Sciences, Riyadh, Kingdom of Saudi Arabia.

**DOI:** 10.7759/cureus.48784

**Published:** 2023-11-14

**Authors:** Abdul Salam Thekkiniyakath Ali, Sultan Nuri Alohali, Yousef Hassan Alheraisi, Bader Abdullah Aldakhil, Sultan Abdulrazaq Alnafisah, Faisal Ibrahim Alsaad

**Affiliations:** 1 Preventive Dental Sciences Department, College of Dentistry, King Saud bin Abdulaziz University for Health Sciences, King Abdullah International Medical Research Center, Ministry of National Guard Health Affairs, Riyadh, SAU; 2 Preventive Dental Sciences Department, College of Dentistry Riyadh Elm University, Riyadh, SAU

**Keywords:** students, saudi arabia, awareness, dentistry, ozone therapy

## Abstract

Background

The field of dentistry is evolving as a result of the increased utilization of contemporary scientific knowledge in dental procedures. When compared to traditional medical treatments like antibiotics and disinfectants, ozone therapy is more budget-friendly, reliable, and conservative. Ozone therapy has proven more effective than the standard treatments available today. Ozone treatment shortens treatment time and eradicates bacterium counts. The fact that there is no discomfort involved with the procedure also helps to boost patient acceptance. Clinical research on the use of ozone in dentistry is limited compared to the amount of research conducted in laboratories. The intent is to assess the knowledge and awareness about ozone dentistry among dental students at King Saud bin Abdulaziz University for Health Sciences, Riyadh, Saudi Arabia.

Materials and Methods

In this examination, a cross-sectional study was undertaken using a questionnaire that has been pre-validated and pre-tested. A total 201 individuals of BDS graduates took part in the survey. Non-probability convenience sampling was used for selecting the samples. The IBM SPSS Statistics, version 25.0 (IBM Corp., Armonk, NY), was used to statistically analyze the collected data. Descriptive statistics in the form of frequencies and percentages were used. To determine the degree of significance, Pearson’s chi-squared test was used; results were deemed significant if p < 0.05. Using Lawshe’s method, the questionnaire’s validity was examined.

Results

The study population included 65 men (48.87%) and 68 females (51.12%), with 22.75 ± 0.76 as the population’s mean age. The research included 51.87% (69) third-year students and 48.12% (64) final-year students. In all, 53.38% knew about dental ozone. Meanwhile, 82.70% of respondents believed that the primary chemical characteristic of ozone is its oxidative nature (p = 0.021), and 78.19% utilized aqueous ozone for hospital disinfection (p = 0.008). With a p = 0.007, 36.84% recognized that ozonated oil is an intra-canal dressing or medicine. Moreover, 37.59% and 42.85% assess gaseous ozone’s microbicidal and wound-healing properties accordingly (p = 0.037, statistically significant). The correlation was calculated using Pearson’s test with a high level of significance between age and gender as well as gender and awareness (p = 0.01). A statistically significant relationship was found between age and awareness (p = 0.036).

Conclusion

The clinical application of ozone is a minimally invasive technique for the prevention and treatment of dental disorders. In order to establish ozone therapy as a widely accepted treatment modality in dental practice, it is necessary to do additional research in the field of dentistry. This research should involve controlled clinical studies that have an adequate follow-up duration and utilize standardized metrics. Although ozone therapy has been demonstrated to be effective, its utilization in the field of dentistry is not commonly practiced.

## Introduction

Ozone, a naturally occurring gas composed of three oxygen atoms, possesses a remarkable property: its ability to filter ultraviolet (UV) light. This property plays a critical role in maintaining the biological equilibrium of the biosphere [1,2]. The term “ozone” derives from the Greek word “ozein,” meaning “to smell,” and it characterizes the gas’s distinct odor detectable by the human nose at concentrations as low as 0.02-0.05 parts per million (ppm) [3]. Ozone therapy, attributed to German chemist Christian Friedrich Schonbein (1840) from the University of Basel in Basel, Switzerland, has been under investigation for over a century. It has numerous applications, including operation room sterilization, blood purification, disinfection, and wound healing in dental surgeries [4,5].

Globally, ozone therapy is gaining recognition as a significant treatment modality, and it has become an integral aspect of dental care in various countries, particularly in Europe and South America [6,7]. Owing to its notable attribute of having the highest oxidation potential, ozone, which is an allotropic form of oxygen, has been employed effectively in numerous dental applications [8]. This is primarily due to its extensive range of uses and beneficial impact on patient care. Among its many applications are potent disinfection, bleeding control, wound healing, antibacterial and immune-stimulating actions, and anti-hypoxic effects [9-11]. However, studies indicate that awareness of ozone therapy in dentistry remains relatively low among both dental professionals and students, with a significant number being unaware of its benefits [11,12]. Conversely, some studies have also reported high levels of awareness among certain professionals, signaling the potential for improvement [11,12].

Hence, the aim of this study is to assess the level of knowledge and awareness of ozone dentistry among dental students at King Saud bin Abdulaziz University for Health Sciences in Riyadh, Saudi Arabia. The findings will help identify areas in need of increased awareness and understanding of ozone therapy. This, in turn, could lead to broader adoption of this treatment modality in dental practice. Ultimately, these efforts will contribute to the enhancement of dental care and the improvement of oral health outcomes for patients.

## Materials and methods

For this cross-sectional study, a self-reported questionnaire, previously validated and pretested through another investigation conducted across objectives similar to ours, was employed [12]. The questionnaire was administered through an online Google form and comprised 15 items, divided into two sections. Section A addressed demographic information, while section B focused on the level of awareness and understanding of ozone therapy applications among dentistry students. The participants were undergraduate students in their third and final years of study for the academic year 2022-2023. The King Abdullah International Medical Research Centre (KAIMRC) provided ethical approval for the study (IRB/2066/23; SP23R/172/07).

The eligibility criteria specified that participants must be undergraduate dental students in their third and final years during the academic year 2022-2023. Inclusion criteria required that participants voluntarily elected to participate in the survey. Exclusion criteria consisted of students not in their third and final years during the academic year 2022-2023 and those who opted not to participate by declining to complete the survey. These criteria ensured the study’s relevance to the target population and upheld the voluntary nature of participation.

The study employed a non-probability convenience sampling method, drawing from a student list provided by the corresponding college. The estimated sample size was 133, derived using the finite population sample size formula with a total population of 201. Participation in the study was voluntary, and consent was obtained upon survey completion and submission. To ensure anonymity and data confidentiality, each participant was assigned a unique identification number. The sample size was determined using the formula n = E2 × Z2 × P × (1 − P), where n represents the sample size, E is the total number of third- and final-year dental students (201), P is the proportion (50%), Z is the statistic (1.95), α is alpha (0.05), and d is the error (0.05).

The IBM SPSS Statistics, version 25.0 (IBM Corp., Armonk, NY), was used to statistically analyze the collected data. Descriptive statistics in the form of frequencies and percentages were used. To determine the degree of significance, Pearson’s chi-squared test was used; results were deemed significant if p < 0.05. Using Lawshe’s method, the questionnaire’s validity was examined, and the results were calculated as follows as CVR = (Ne − N/2)/(N/2), in which CVR is the content validity ratio, Ne is the number of panelists indicating “essential,” and N is the total number of panelists.

## Results

The study population consists of 65 males (48.87%) and 68 females (51.12%). The mean age of the population is 22.75 ± 0.76 (mean ± SD). In all, 51.87% (69) of students from D3 (third-year BDS undergraduate students) and 48.12% (64) from D4 (fourth-year BDS undergraduate students) participated in the study. The responses toward awareness are illustrated in Table 1, along with the chi-square analysis.

**Table 1:** Awareness of respondents on ozone therapy among third- and final-year undergraduate students

Variables	Responses	Frequency, n (%)	Chi-square	p-value
Do you have knowledge about dental ozone therapy?	Yes	62 (46.61)	5.992	0.068
	No	71 (53.38)		
The distinctive chemical characteristic of the ozone layer can be ascribed to its?	Oxidative	110 (82.70)	4.320	0.021
	Non-oxidative	13 (9.77)		
	Reductive	10 (7.51)		
Ozone is a molecule with a triangular configuration?	Tetra-oxygen	15 (11.27)	2.095	0.139
	Tri-oxygen	4 (3.00)		
	Di-oxygen	114 (85.71)		
The type of ozone used to disinfect dental hospitals?	Gaseous	29 (21.80)	0.126	0.008
	Aqueous	104 (78.19)		
What is the specific type of ozone used in the field of dentistry?	Ozonated Oil	6 (4.51)	6.309	0.005
	Ozonated water	17 (12.78)		
	Both ozonated oil and water	86 (64.66)		
	Neither	24 (18.04)		
Can ozonated water effectively decrease the quantity of ---- present on the surface of the denture base?	Candida albicans	34 (25.56)	0.751	0.409
	Streptococcus mutans	99 (74.43)		

More than half of respondents knew about ozone in dentistry (53.38%). The distinctive chemical property of ozone has been reported as oxidative by 82.70% along with statistical significance (p = 0.021), and 78.19% opted for an aqueous form of ozone that is used for disinfection in hospitals with a high significance value (p = 0.008). The majority responded that Streptococcus mutans in denture plates can be reduced by ozonated water. High significance with p = 0.005 was estimated for the query regarding the form of ozone used in dentistry.

The feedback on knowledge about ozone dentistry provided by the respondents is represented in Table 2.

**Table 2:** Knowledge about ozone in dentistry

Variables	Responses, n (%)			Chi-square	p-value
	Yes	No	Not aware		
Does the aqueous ozone form possess appropriate cell biological characteristics for oral application?	62 (46.61)	2 (1.5)	69 (51.87)	0.853	0.014
Can ozone relieve pain and reduce inflammation?	48 (36.09)	5 (3.75)	80 (60.15)	7.259	0.082
Does ozonated nanobubble water have bactericidal effects?	57 (42.85)	2 (1.5)	74 (55.63)	3.561	0.160
Is it possible to use ozonated oil as an intra-canal dressing/medication?	49 (36.84)	7 (5.26)	77 (57.89)	1.945	0.007
Gaseous ozone has more effective microbicidal properties?	50 (37.59)	10 (7.51)	73 (54.88)	4.059	0.904
Ozone therapy leads to faster wound healing?	57 (42.85)	7 (5.26)	69 (51.87)	1.988	0.037

The majority of the participants were not aware (51.87%) that the aqueous form of ozone fulfilled optimal cell biological characteristics in terms of oral application, with p = 0.014 exhibiting high significance. The preponderance was neither aware of the analgesics and anti-inflammatory action (60.15%) nor that ozonated nanobubble water has bactericidal effects (55.63%). In all, 36.84% knew about the use of ozonated oil as an intra-canal dressing or medication, with a high statistical significance (p = 0.007). Moreover, 37.59% and 42.85% knew the effective microbicidal action of the gaseous ozone and its faster wound healing property (p = 0.037, statistically significant), respectively.

The degree of correlation that exists between different factors, such as age, gender, and level of awareness, was calculated using Pearson’s test (Table 3).

**Table 3:** Estimating the correlation between the variables

Variables	Pearson's correlation	p-value
Age and gender	0.861	0.004
Age and awareness	0.295	0.036
Gender and awareness	0.502	0.001

It was found that there was a high level of significance between age and gender, as well as gender and awareness (p = 0.01). The obtained value of 0.295 suggests a statistically significant association between age and awareness (p = 0.036).

## Discussion

Ozone therapy has a long-standing history as a therapeutic intervention for an array of medical conditions [12]. The outcomes of our research provide insights into dentists’ understanding of ozone therapy as an alternative to conventional treatments. We found that a considerable proportion of the survey respondents (74.5%) were unaware of the potential applications of ozone in dentistry. Conversely, a lesser percentage (25.5%) demonstrated knowledge of ozone therapy’s use in dentistry. This outcome aligns with previous research by Hannah (2016) [13], where 37 out of the total participants (36.6%) claimed awareness of ozone therapy in dentistry, while the remaining 64 participants (63.4%) professed ignorance.

Our study provided more specific figures, showing that while 46.61% of participants were informed about ozone dentistry, 53.38% were not. In our sample, 25.5% of individuals acknowledged the antibacterial properties of ozone therapy, and a slightly higher percentage (37.59%) responded similarly [14]. We observed a statistically significant association between the respondents’ gender and their understanding of ozone’s effectiveness in combating harmful bacteria. Our findings (p = 0.904) are in agreement with the p-value of 0.507 (>0.05) [15], which is statistically insignificant. In response to inquiries about ozone therapy’s chemical properties, 82.70% of respondents identified the unique chemical characteristic of ozone as being oxidative, a result that also demonstrated statistical significance (p = 0.021). Similarly, Pushpaanjali et al. (2020) [16] reported that 69.9% of survey participants recognized the primary chemical feature of the ozone layer as oxidative, in comparison to 22.3% who identified it as non-oxidative and 7.8% who classified it as reductive.

A statistically significant correlation was observed between age and knowledge of ozone therapy usage. Among undergraduate students, 5.83% identified ozone as reductive, while postgraduate students reported 1.94% reductive [16]. Furthermore, a Pearson test p-value of 0.001 indicated that 59.22% of undergraduates and 10.68% of postgraduates recognized oxidative properties, compared to 15.53% of undergraduates and 6.80% of postgraduates who identified non-oxidative properties [16]. This is consistent with our current investigation, which suggests a statistically significant correlation between awareness of ozone therapy and age (p = 0.036), as indicated by the obtained value of 0.295.

In this study, we found that 42.85% of respondents, a statistically significant segment of the population, recognized ozone’s potential to accelerate wound healing. This aligns with previous findings that ozone promotes the synthesis of physiologically active substances, such as prostaglandins and interleukins, which are renowned for their pivotal role in mitigating inflammation [17]. Although our research did not detect statistical significance in the anti-inflammatory benefits of ozone therapy, our results provide additional evidence supporting these effects. The efficacy of ozonated water in reducing Candida albicans on denture plates has been demonstrated, particularly when dentures were immersed in ozonated water at a concentration of 10 parts per million (ppm) for a duration of 30-60 minutes [18]. A previous investigation by Murakami et al. (1996) [19] suggested that ozone gas was superior to ozonated water in eliminating C. albicans.

In a separate study by Al Habashneh et al. (2015) [20], the impact of a one-minute exposure to ozone, in both its aqueous and gaseous states, was assessed for its potential benefits in inhibiting periodontal pathogens such as Tannerella forsythia, Porphyromonas gingivalis, and Parvimonas micra. Notably, the high oxidation potential of ozone on calcium-coated surfaces [21] results in the formation of calcium oxalate, a compound often used in desensitizing agents designed to coagulate or seal dentinal tubules [22]. This characteristic further substantiates the unique chemical property of ozone. Our research findings present a high degree of statistical significance, supporting the potential application of ozonized oil as an intra-canal medication [23]. Prior to ozone administration, it is crucial to exclude certain conditions, including severe myasthenia, pregnancy, hyperthyroidism, severe anemia, recent myocardial infarction, acute alcohol intoxication, organ hemorrhage, and ozone allergy [24].

Although the sample size of 201 participants was adequate for certain analyses, it may have limited the detection of more nuanced variations or correlations within the population, which is a potential limitation of this study. A broader and more diverse sample could provide a more comprehensive understanding of awareness and knowledge. Furthermore, the research’s reliance on a single institution may have narrowed the range of experiences and perspectives considered. Future multi-center studies encompassing several dental schools or medical facilities could offer a more extensive understanding of ozone therapy awareness and use.

## Conclusions

The evolution of science has progressively transformed dentistry, setting new patient expectations for pain-free care. However, a certain degree of misunderstanding persists among some dentists regarding the benefits of ozone therapy. The application of ozone therapy has elevated the standard of care and has streamlined treatment approaches, yielding significant advantages for patients. This study reveals that dentists possess a considerable understanding of ozone therapy and its associated benefits. Despite being a validated method, ozone therapy is infrequently employed in dentistry, even though it has been identified as having a broad range of applications within the field. To counteract this lack of awareness, dental education programs and professional organizations can adopt an active role in providing supplementary training and disseminating information about ozone therapy. Moreover, the sharing of successful case studies and research findings within the dental community can act as a catalyst for augmenting awareness. This could inspire dentists to explore the advantages of incorporating ozone therapy into their dental practices.
